# Evolution of Consciousness

**DOI:** 10.3390/life14010048

**Published:** 2023-12-27

**Authors:** Danko D. Georgiev

**Affiliations:** Institute for Advanced Study, 30 Vasilaki Papadopulu Str., 9010 Varna, Bulgaria; danko@q-bits.org

**Keywords:** brain cortex, causal potency, conscious experience, quantum physics, sentience

## Abstract

The natural evolution of consciousness in different animal species mandates that conscious experiences are causally potent in order to confer any advantage in the struggle for survival. Any endeavor to construct a physical theory of consciousness based on emergence within the framework of classical physics, however, leads to causally impotent conscious experiences in direct contradiction to evolutionary theory since epiphenomenal consciousness cannot evolve through natural selection. Here, we review recent theoretical advances in describing sentience and free will as fundamental aspects of reality granted by quantum physical laws. Modern quantum information theory considers quantum states as a physical resource that endows quantum systems with the capacity to perform physical tasks that are classically impossible. Reductive identification of conscious experiences with the quantum information comprised in quantum brain states allows for causally potent consciousness that is capable of performing genuine choices for future courses of physical action. The consequent evolution of brain cortical networks contributes to increased computational power, memory capacity, and cognitive intelligence of the living organisms.

## 1. Introduction

Humans are *sentient* beings, who access the surrounding physical world through their *conscious experiences* [1,2,3]. The sensory flow of information from the surrounding world and from our own body to the *brain cortex* informs us about the physical reality we live in and the physical abilities of our body. The accumulation of memories about past experiences helps us accumulate knowledge and develop a sense of *selfhood* before we reach our second year in life [4]. We share this sense of selfhood and self-recognition with other animals, including great apes [5], monkeys [6], elephants [7], dolphins [8], and cleaner fish [9]. This convergent evolution of cognitive abilities in such a diverse group of animals suggests that *sentience* is already present at the time of our last common ancestor with great apes about 13 million years ago [10], with monkeys about 20 million years ago [11], with elephants about 60 million years ago [12], with dolphins about 95 million years ago [13], and with fish about 375 million years ago [14]. Our evolutionary history may be traced even further back in time to the last common ancestor of humans with other living organisms, which is a single cell about 3.8 billion years ago [15,16,17,18,19], and ingenious chemical experiments have revealed that eventually the first life forms could have arisen from non-living matter under primitive earth conditions [20,21].

The name of our species *Homo sapiens*, translated from Latin as “man the wise”, emphasizes the role of human mental capabilities for the development of stone tools, mastering fire, venturing out of Africa, and eventually populating all seven continents on Earth [22,23,24]. The evolution of human consciousness, with its characteristic features of intelligence, creativity, and innovation, has been customarily inferred from the steadily increasing cranial volume of ancestral species. The average cranial volume is 441 cm^3^ in *Australopithecus africanus* who lived 4–2 million years ago [25], 640 cm^3^ in *Homo habilis* who lived 2.3–1.5 million years ago [25], 937 cm^3^ in *Homo erectus* who lived 2–0.8 million years ago [25], 1206 cm^3^ in *Homo heidelbergensis* who lived 0.7–0.2 million years ago [26], and 1350 cm^3^ in modern *Homo sapiens* who originated 0.3 million years ago [25]. Importantly, the evolutionary advantage of hominid species resulted not from the larger cranium itself, but from the enhanced cognitive abilities and newly evolved mental features, manifested in the form of language, empathy, cultural traditions, and moral values [24,27,28]. We know that our human consciousness has evolved naturally through comparative studies with other animal species. Yet, we are the first species whose *consciousness* no longer relies solely on *genes* for reproduction and transmission in future generations, but may also utilize recorded *ideas* that were discovered by previous generations and stored as written text in printed books or as visual images captured in sculptures, photographs, or art.

The main evolutionary problem of consciousness is that *sentience* cannot miraculously emerge out of *insentient* matter. In fact, the mathematical nature of physical laws is such that nothing *qualitatively different* can emerge out of physical equations that contain only classical physical quantities such as mass, charge, length, and time. Consciousness can be *causally potent* only if it is already present in some form inside the physical equations [29,30]. Natural scientists at the end of 19th century were already aware of the latter problem of *mental causation* and have derived the incompatibility of emergent consciousness with the Darwinian biological evolution of consciousness; if the emergent consciousness is constrained due to mathematics to be causally impotent in the physical world then such a causally impotent entity cannot provide any benefit to the organisms that possess it, and consequently cannot be selected for by natural selection [31,32,33,34].

The failure of emergent consciousness was not instantly recognized, despite heated debates at the end of 19th century. Rather than concluding that there is something wrong with the idea of *emergence*, natural scientists attributed the problem to the idea of *causality* or the idea of *physicality*. For example, *epiphenomenalism* is one philosophical stance that entertains the dubious idea that our consciousness is a causally impotent *epiphenomenon* that indeed is evolutionary useless and provides no benefits to those organisms that have consciousness [35]. Alternatively, *nonphysicalism* is another philosophical stance that entertains the dubious idea that the mind operates miraculously outside the physical laws and could survive even if it is not supported by a physical substrate [36].

Here, we will defend a modern viewpoint based on quantum physics according to which *sentience* is a fundamental property of elementary physical particles, implying that causally potent animal consciousness evolved from causally potent sentient matter [1,2,3]. In Section 2, we discuss the importance of prehistoric art as evidence that our conscious experiences are indeed causally potent and able to exert a tangible impact on the surrounding physical world. In Section 3, we describe the relationship between physicalism and sentience. In Section 4, we compare the brain size in different mammal species and link the anatomical complexity of their brain cortex with higher cognitive functions. In Section 5, we explain how the physical properties of the sentient brain are a manifestation of the quantum dynamics of elementary physical particles that comprise all biomolecules. In Section 6, we conclude with a brief summary of how the quantum revolution in physics has profoundly changed our understanding of consciousness and its place in the physical world. Lastly, in Section 7, we provide a comprehensive glossary of the main terms and concepts that are used repeatedly throughout this work.

## 2. Causal Potency of Conscious Experiences Is Manifested in Prehistoric Art

The essence of consciousness is in the private phenomenological character of conscious experiences. For example, when we look at the blue sky or when we inhale the salt air from the ocean, there is a particular *feeling* that we experience for each situation, and there is no particular behavior that is expected from us. Consequently, our consciousness is not a kind of behavior or some form of action, but rather a state of existence, which we refer to as a *sentient state* or a *mental state*. This mental state changes dynamically in time, creating a flow of feelings that comprise the *stream of conscious experience*.

**Figure 1:**
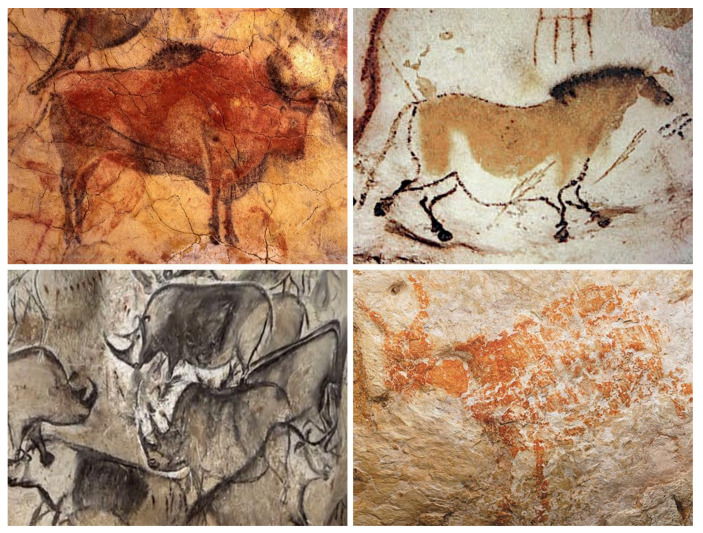
Prehistoric cave paintings depicting different animals as consciously perceived by their prehistoric painters. Top left: Polychrome bison from the Cave of Altamira in Cantabria, Spain, dated to the Magdalenian culture circa 14,000 years ago. Top right: Painting of a horse from the Lascaux Cave in Montignac, France, dated to circa 19,000 years ago. Bottom left: Painting of rhinos from the Chauvet Cave in Vallon-Pont-d’Arc, France, dated to circa 32,000 years ago. Bottom right: Painting of a bull from Lubang Jeriji Saléh in Borneo, Indonesia, dated to circa 40,000 years ago. The images of prehistoric work of art are in the public domain.

The supposition that our consciousness is an epiphenomenon lacking any causal potency upon the physical world is self-defeating and contradicts available experimental evidence, most notably in the form of prehistoric art. The primary purpose of art is to capture the conscious experiences of the artist and then to evoke similar conscious experiences in the observer [37]. Undoubtedly, for prehistoric artists, the most important aspect of their lives were the animals that were hunted and provided much needed meat for food and skins for clothing during the harsh ice age conditions [38]. In order to convey this knowledge to future generations, the prehistoric artists were painting their caves with exquisite images of bison, horses, rhinos, or bulls (Figure 1).

The Cave of Altamira in Cantabria, Spain, was discovered in 1868 by Modesto Cubillas (1820–1881) and studied by Marcelino Sanz de Sautuola (1831–1888), who attributed the cave paintings to Paleolithic artists [39]. After initial public acclaim, the findings were rejected as forgeries by contemporary experts, including Émile Cartailhac, according to whom prehistoric human beings lacked sufficient ability for abstract thought [40]. The reputation of de Sautuola was cleared only posthumously by Cartailhac, who published an apology in 1902 [41]. Modern dating techniques have confirmed that the earliest drawings in the Cave of Altamira are from 35,600 to 22,000 years ago [42], while the iconic Magdalenian polychrome bison was dated to circa 14,000 years ago [43].

The discovery of the Lascaux Cave in Montignac, France, by Marcel Ravidat on 12 September 1940, has further revealed hundreds of prehistoric art paintings and engravings, which depicted animals, human figures, and abstract signs, some of which were dated from 23,500 to 17,000 years ago [44,45]. The Chauvet Cave in Vallon-Pont-d’Arc, France, discovered by Eliette Brunel-Deschamps, Christian Hillaire, and Jean-Marie Chauvet on 18 December 1994, contains exquisite paintings of lions, horses, bison, and rhinos, dated to Aurignacian culture from 32,000 to 30,000 years ago [46,47,48,49]. The oldest known prehistoric painting, dated to circa 40,000 years ago, depicting a bull made in ochre, was discovered in the Lubang Jeriji Saléh cave, East Kalimantan, Borneo, Indonesia [50]. Taken together, the prehistoric paintings of animals establish that the conscious experiences of the prehistoric painters perceiving those animals were indeed causally potent in leaving tangible marks of paint on the cave walls and ceilings.

**Figure 2:**
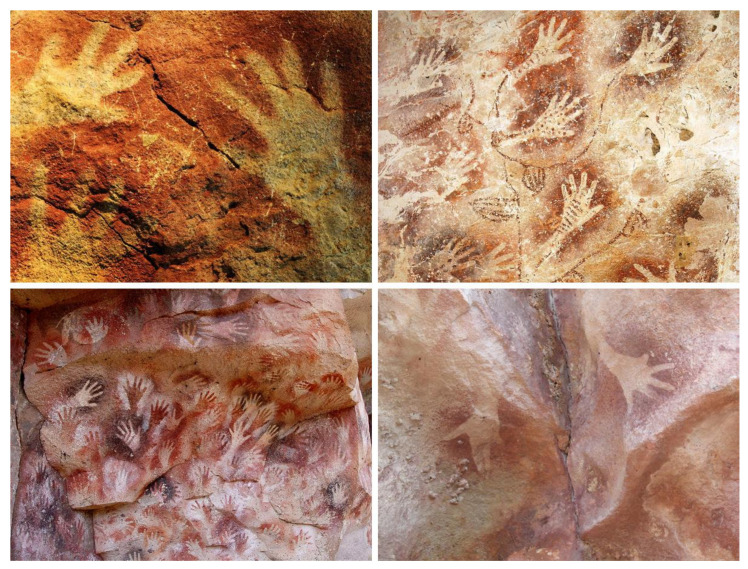
Prehistoric hand stencils communicating the identity of their prehistoric painters. Top left: Handprints created by blowing ochre mixed with water over the hands from the Cave of Altamira in Cantabria, Spain, dated to circa 17,200 years ago. Top right: The tree of life hand prints from Gua Tewet in Borneo, Indonesia, dated to circa 9800 years ago. Bottom left: Hand stencils from Cueva de las Manos in Argentina, dated to circa 9300 years ago. Bottom right: Hand stencils with mutilated little finger at the Djulirri rock art site in the Wellington Range of Arnhem Land, Northern Territory, Australia, dated to circa 7000 years ago. The images of prehistoric work of art are in the public domain.

The cognitive abilities of prehistoric painters undoubtedly exceeded the sensory capacity of their five senses as they were capable of *abstract thought*, including the expression of concepts such as *selfhood* or *personal identity* through colorful hand stencils left on cave walls or rocks (Figure 2). Indeed, the hand stencils are conceptually similar to the practice by illiterate subjects of putting their thumb in ink and then impressing it on paper in order to substitute for their signature on legal documents. Color handprints were found in the Cave of Altamira, dated to circa 17,200 years ago [51], Gua Tewet in Borneo, Indonesia, dated to circa 9800 years ago [52,53], Cueva de las Manos in Argentina, dated to circa 9300 years ago [54], and at the Djulirri rock art site in the Wellington Range of Arnhem Land, Northern Territory, Australia, dated to circa 7000 years ago [55].

The prehistoric painters communicated their identity by what we might call a primitive color “photograph” of their hands, which are the creative tools that transform the surrounding physical world [56]. In fact, modern language still carries the symbolism of the hand as a *certificate* of the identity of its bearer. For example, the expression “never forget the hands that raised you” reminds us to always remember and be grateful to those individuals who generously took care of our upbringing.

Interestingly, the observation of hand stencils with missing fingers across different Paleolithic caves, dated to Gravettian culture from 33,000 to 21,000 years ago, has been interpreted as a type of sign language used for silent rituals or hunting [57,58]. Indeed, it seems likely that the prehistoric hunters, who were endowed with spoken language, would have used silent hand signs to communicate without scaring their prey during a hunt. The numerous hunting scenes with wild animals and hunters [59,60,61] testify that our prehistoric ancestors were *consciously aware* of what they were *doing* and understood what they intended as *documenting* for the future generations. Thus, the wonderful prehistoric art created by our ancestors is reassuring with regard to the *causal potency* of conscious experiences and provides compelling evidence against *epiphenomenalism*.

It is worth emphasizing that the causal potency of consciousness can only be established by theoretical interpretation of experimentally collected data. Observed behavior in itself is not sufficient to establish presence of conscious experiences and/or causal efficacy of those conscious experiences. For example, a computer program may generate art, which we may not be able to attribute to any conscious mind with its own intentionality. In fact, since we are not biologically related to the art generating computer program, there are no immediate considerations that we can further use to deduce presence of computer consciousness. However, the situation is completely different when we interpret the art produced by our human ancestors to whom we are biologically and evolutionary related. Observing the prehistoric art, including communication of identity through hand stencils, is something that we can relate to through our own conscious experiences, and we can easily *empathize* with the prehistoric creators. Thus, from our modern evolutionary point of view, the creation of prehistoric art *is best explained* by the causal potency of consciousness possessed by our prehistoric ancestors. This conclusion is based on the *humble* premise that none of us individually is qualitatively exceptional with regard to the characteristics possessed by other members of the biological species.

The *inference to the best explanation* is a scientific method [62] that does not aim to prove inductively some general statement from finite data, but rather to demonstrate that one general statement performs better in explaining the observable world compared to other rival statements. With regard to the stated causal potency of consciousness, one rival statement is that our prehistoric ancestors did not have any conscious experiences while creating their paintings, yet we miraculously possess conscious experiences. Another rival statement is that our prehistoric ancestors did have conscious experiences of the observed animals, but these conscious experiences were not causally potent in the creation of the paintings of those animals. Both rival statements are *poor explanations* of prehistoric art because they contradict Darwinian evolution of human consciousness through natural selection. In other words, the construction of a plausible evolutionary account of human consciousness necessarily requires the causal potency of conscious experiences.

## 3. Physicalism and Sentience

Theoretical physics is supposed to describe everything that *exists* in the *universe*. Since the universe is the collection of all existing things, and our conscious minds surely do exist, then it logically follows that consciousness is physical and constitutes a valid subject for investigation by theoretical physics [1].

Darwinian evolution through natural selection [63,64] is a process that obeys the physical laws of the universe. This means that living organisms could not miraculously evolve *sentience* if the physical laws state that all physical entities are *insentient*. Furthermore, the very idea of labeling conscious experiences as *nonphysical* is predicated on the erroneous assumption that the list with physical laws is somehow externally given to us and we already know what the ultimate physical theory is. In fact, the list with physical laws is fundamentally different for *classical physics* or for *quantum physics* [1,65]. To be able to meaningfully state that a phenomenon is nonphysical, we should be able to prove that not only all currently available physical theories cannot describe that phenomenon, but also all possible future physical theories will not be able to describe that phenomenon too. Therefore, without access to all possible future physical theories, one cannot meaningfully maintain that consciousness is nonphysical.

The mathematical structure of physical theories consists of a list of *physical postulates* that describe the *physical laws* of the *universe* [66]. *Miracle* is a phenomenon that is impossible to occur or whose occurrence cannot be predicted within the framework of a particular physical theory [67]. The repeated experimental observation of a miraculous phenomenon, however, is not an indication that miracles occur in the universe, but rather is a demonstration that the particular physical theory under consideration is *false* [68,69,70,71]. The theoretical solution is to develop a new physical theory, which can predict the observed phenomenon, thereby no longer classifying it as a miracle. The principle affirming that “miracles do not occur in the universe” is just an alternative formulation of the fact that we are always free to improve and update our physical theories when they are found to be in conflict with experimental observations [72,73].

The problem of *mental causation* is specific to the particular conceptual combination of *emergent consciousness* and *classical physics*. The principles of classical physics, according to which all physical entities are *observable* and undergo *deterministic dynamics* governed by ordinary differential equations, imply that emergent unobservable conscious experiences cannot have any causal effects on the surrounding physical world [29]. Without causal potency, emergent sentience in classical physics is epiphenomenal, cannot confer any advantage or disadvantage to organisms that possess it, and consequently cannot evolve through natural selection. Therefore, the only way forward toward a theoretical description of the evolution of consciousness is a *reductive* modification of the physical theory and incorporation of *sentience* as a fundamental ingredient in the physical laws that govern the properties of physical reality. Fortunately, *classical physics* has already been found to be experimentally inadequate and was replaced in 1920s with newly discovered *quantum physics*. The founding fathers of quantum physics, including Max Planck [74,75,76,77], Albert Einstein [78,79], Louis de Broglie [80,81], Erwin Schrödinger [82,83], Paul Dirac [84], John von Neumann [85,86] and Max Born [87,88], have already accomplished the hard work of axiomatizing quantum theory and characterizing those quantum features, which underlie the success of quantum theory as a faithful description of physical reality. Currently, our task is to apply quantum theory to biological systems.

Among the most important features of quantum physics is that what exists in the physical world is not what can be observed, namely, *quantum states* are *unobservable*, whereas *quantum observables* are actual *choices* performed by the quantum systems at the time of their measurement [2,3]. Consequently, if conscious experiences are reductively identified with the quantum information contained in the unobservable quantum states, the resulting quantum physical theory of consciousness will imply that *sentient* brains have evolved from *sentient* matter. Thus, a reductive physical theory of sentience no longer requires the dubious concept of emergence. The evolutionary psychologist William James expressed the latter conclusion quite eloquently in his 1890 textbook:

“We ought therefore ourselves sincerely to try every possible mode of conceiving the dawn of consciousness so that it may not appear equivalent to the irruption into the universe of a new nature, non-existent until then. […] *If evolution is to work smoothly, consciousness in some shape must have been present at the very origin of things*.” [32].

## 4. Brain Size and Cognitive Abilities in the Evolutionary Tree of Life

The anthropocentric view that biological evolution is a hierarchy of complexity with humans on top is deeply misguided, and acutely problematic to the extent of it being a fallacy [89,90,91]. The technological achievements of humanity for supporting life in inhospitable environment and landing on the moon [92] are impressive, but do not justify the division of living organisms into “higher” and “lower”. All modern non-human animal species are well adapted to their habitat and enjoy a particular way of life, which means that they should not be viewed as “failed wannabe humans”. Furthermore, if one considers physics seriously, it is clear that the physical composition of the human brain contains exactly the same chemical atoms and elementary particles that can be found in the surrounding non-living world [29]. Because the characteristic properties of elementary particles remain the same regardless of whether they comprise a brain or not, it follows that the problem of *sentience* is a subject to theoretical physics and has to be resolved by the nature of physical laws. Then, the evolution of sentient brain from sentient matter can improve the computational capacity of the brain neural networks [93] or increase their memory storage capacity [1], but in terms of quantitative anatomical measures we as humans have neither the largest brain, nor the most convoluted brain cortex [94].

**Figure 3:**
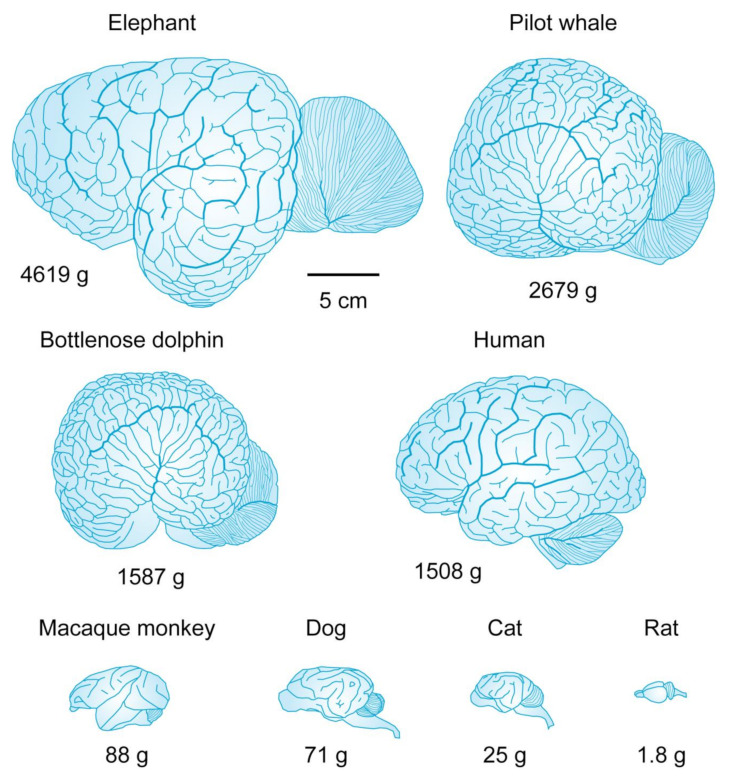
Brains of different modern mammals, viewed from the side. Humans do not have the largest brain size, as evident from the average brain mass of elephants, whales, and dolphins. Furthermore, the human brain cortex does not have as many cortical convolutions and surface area as dolphins and whales do. Although humans are intelligent and have achieved remarkable control over the surrounding world, they are not situated “higher” than other animals in the evolutionary tree of life. The brain size is fully appropriate to the particular habitat and lifestyle enjoyed by each animal.

The average *brain mass* of the human (*Homo sapiens*) is 1508 g [95], which is larger than macaque monkey (*Macaca mulatta*) 88 g [96], dog (*Canis familiaris*) 71 g [97], cat (*Felis catus*) 25 g [96], and rat (*Rattus norvegicus*) 1.8 g [95], but is smaller than African elephant (*Loxodonta africana*) 4619 g [98], pilot whale (*Globicephala macrorhynchus*) 2679 g [99], and bottlenose dolphin (*Tursiops truncatus*) 1587 g [100] (Figure 3).

Similarly, the average *total surface area* of the brain cortex in humans is 2430 cm^2^ [101], which is larger than macaque monkey 250 cm^2^ [101], dog 103 cm^2^ [97], cat 83.3 cm^2^ [101], and rat 6.44 cm^2^ [101], but is smaller than African elephant 6275 cm^2^ [101], pilot whale 5815 cm^2^ [101], and bottlenose dolphin 3745 cm^2^ [100]. Because the brain cortex is the seat of higher cognitive functions, it is not surprising that elephants and dolphins demonstrate remarkable intelligence and easily cover objective criteria for non-human *personhood*, namely, they are alive, aware of their environment, have emotions, possess individual personalities, exhibit self-control, and treat others appropriately, even ethically [102].

Dolphins are an excellent example of intelligent social animals. They live in tightly-knit social groups, communicate with each other using a vast array of sounds and nonverbal gestures, and interact with other species, including people [103]. Dolphins can carry on conversations in an advanced spoken language made up of pulses and whistles [104]. Interestingly, the dolphins do not interrupt each other, which suggests that each dolphin listens to the other’s pulses before producing its own [104]. Similar to humans, dolphins appear to have individual names. For example, bottlenose dolphins produce *signature whistles* to identify themselves amongst groups [105]. Dolphins can also produce *echolocation clicks* used for hunting and feeding [106,107,108,109], *buzzes* used for social interaction and mating [110], and *burst-pulsed sounds* used when fighting or defending against predatory threats [111,112,113]. Remarkably, dolphins are *altruistic* creatures and could adopt orphaned calves from other delphinid species [114]. Dolphins are also famous for rescuing humans in distress at sea, either by allowing drowning humans to ride on their back or by psychologically encouraging them with their presence to continue their efforts to reach the shore [115]. Dolphins have also saved unsuspecting human surfers or swimmers on multiple occasions by warding off aggressive Great White sharks [116,117,118]. This unsolicited friendly behavior toward us is to be contrasted with the long-held human tradition of *mass dolphin slaughter* in certain North Atlantic islands, which is defended absurdly by their local government with “the abundance of whales, dolphins, and porpoises in their waters” [119] completely ignoring the cruelty of the act of slaughtering the dolphins by severing the main blood supply to the brain and the main nervous system [120,121,122]. Sadly, dolphin and whale hunting is still not illegal in all countries, attesting to the fact that humanity needs to put more efforts toward improving its moral stance on animal exploitation [123,124,125] and/or environment preservation [126,127,128].

## 5. Quantum Substrates Inside Neural Tissue of Living Organisms

Quantum physics is the most successful, experimentally corroborated, modern physical theory explaining what is *real*, what is *observable*, and what is *possible* [1]. The most important departure from the deterministic clockwork world of *classical physics* is the introduction of quantum *potentialities* and quantum *actualities* represented by two fundamentally different mathematical objects, namely, *state vectors* and *observable operators* on Hilbert space [1,2,3]. This latter mathematical distinction implies that *what exists* is not the same as *what can be observed* in the quantum world [129,130,131].

The quantum *state vectors* are physical solutions of the Schrödinger equation and represent *what exists* in the form of *quantum probability amplitudes* [82,83,84,85]. These quantum probability amplitudes are then subject to the Born rule [87,88], which produces physical *potentialities* in the form of *quantum probabilities* for different future courses of action. The *actualization* of a particular course of action is represented by a *quantum jump*, during which the quantum state vector of the quantum system undergoes stochastic transition from a linear superposition of eigenvectors to a single eigenvector of the measured quantum *observable operator* [132,133,134,135,136]. Thus, the mathematics of quantum stochastic transitions affirms that quantum systems are indeed capable of making *genuine choices*, thereby exhibiting their own *free will* [29,30].

*Free will* is the inherent capacity of physical agents to perform genuine *choices* among at least two, but often more, available future physical outcomes [1,29,30]. In classical deterministic physics, the future is fully determined by the present, which means that free will is impossible due to the fact that there is only a single future physical outcome available, thereby precluding any choosing [1,29,30]. Consequently, a number of classical redefinitions of free will have been proposed based on effective *unpredictability* of future courses of action based on the extreme complexity of the brain neural networks, including nonlinear interactions and possible occurrence of *deterministic chaos* [137,138,139,140]. Here, we emphasize that redefinition of free will as *unpredictability* of future behavior [141] is irrelevant for moral judgement and blame attribution because, for example, when someone commits a crime, the main consideration is whether the person *could have done otherwise* and not whether the crime could have been predicted or not. Therefore, besides *sentience*, the focus throughout this work is put on the *capacity of making genuine physical choices*, regardless of whether it is called “free will” or not. Determinism in classical physics forbids any such making of genuine physical choices. Fortunately, in quantum indeterministic physics, quantum systems possess the capacity to genuinely choose from several available future physical outcomes. Furthermore, it can be proven that if human experimenters possess free will, i.e., have the capacity to choose, then the measured quantum particles also possess free will, i.e., also have the capacity to choose [142,143]. This is a logically consistent result because it becomes possible to explain where the postulated human free will comes from, namely, the human anatomical brain is made of quantum particles thereby harnessing the free will of its quantum constituents [1,29,30]. Thus, quantum physics naturally leads to a form of *panpsychism* or *panexperientialism*, upon which we will elaborate next.

The physical *act of choosing* could be described by stochastic differential equations and Itô calculus [29]. At certain times when quantum systems interact strongly with their physical environment, the quantum state could be acted upon with *projection operators*, which implement *quantum measurement* of some quantum physical observable [85]. In quantum physics, not all physical observables could be measured simultaneously [144]. The measured quantum observable presents a *question* being asked to the observed quantum system, and this question could be set by a human experimenter using suitable measuring device. The human experimenter, however, cannot choose on behalf of the measured quantum system what the *answer* to the posed question will be. Upon quantum measurement, the observed quantum system is presented with available future physical outcomes, referred to as *eigenvalues* of the measured *quantum observable*, which can be realized with certain quantum probabilities. Then, the quantum system acts as an agent and chooses to actualize one particular physical outcome, thereby converting the quantum probability of this actualized outcome to unity for all future times, while simultaneously zeroing the quantum probability of all other rejected physical outcomes. Mathematically, the act of choosing performs a *quantum jump* so that the dynamic trajectory of the quantum system may not be smooth, but rather be stochastic [145,146,147]. In the act of choosing, the quantum probabilities play the role of inherent *biases* or *desires* of the quantum agent [30]. When the probabilities are equal for all available physical options, the choice is completely unbiased. However, quantum systems can also make biased choices if one probability is larger than the others. Classically, there is no room for biases as physical outcomes either occur or do not occur with absolute certainty. In quantum physics, however, inherent biases and genuine choices are possible and intimately related to the process of learning [29,30].

*Sentience* and *free will* are two distinct physical properties that do not have to be instantiated together. Nevertheless, sentience without free will deprives life from meaning because conscious minds would be experiencing a streaming *life story* that is beyond their control, resembling very much the situation of a spectator in the cinema who cannot choose the ending of the movie that is being played. Conversely, free will without sentience deprives the act of choosing from meaning because the physical agent would not have a conscious mind to care about the consequences of the chosen physical outcomes. The combination of sentience and free will is the only one that makes life worth living, because each conscious agent would be at least partially responsible for the potential happy ending of their own life story. Partial responsibility arises from the fact that inside a universe full of interacting physical agents, each of which is endowed with free will, one can only control one’s own free actions, but would have to suffer the free actions by others. It is also worth pointing out that the quantum mechanical formalism admits a number of mutually contradicting interpretations, varying from *conspirative superdeterminism* to *multiverse with splitting minds like amoebas* [1]. Each of these interpretations of quantum mechanics has different implications for the causal potency of consciousness or the existence of free will. In this present work, we advocate a quantum reductive approach to consciousness in which both sentience and free will are attributed to elementary physical particles [1,2,3]. As a consequence, the evolution of a sentient brain cortex from sentient particles becomes a natural process that obeys the quantum physical laws without the need of any miraculous emergence of conscious experiences from insentient substrates.

**Figure 4:**
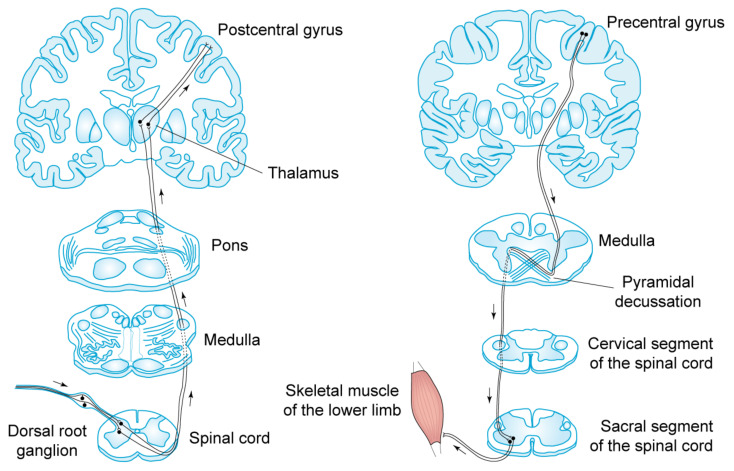
The human brain cortex communicates continuously with its physical environment through electric signals. Left: the somatosensory pathway delivers sensory information from the human body to the somatosensory cortex, which is located in the postcentral gyrus. Right: the somatomotor pathway delivers motor information from the motor cortex, which is located in the precentral gyrus, to the body muscles. The spinal cord segments, medulla and pons are represented with their transversal sections, whereas thalamus and cortex are shown in frontal slice. Modified from Ref. [1].

The *sentient* brain cortex receives *sensory inputs* from the surrounding environment and performs *free choices* that control the body muscles through *motor outputs* in the form of electric signals transmitted along the somatomotor pathway (Figure 4).

**Figure 5:**
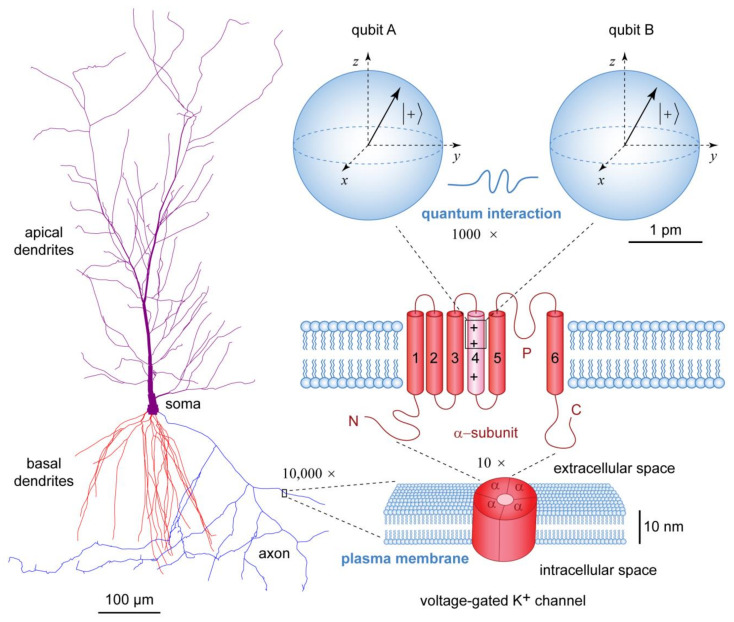
Different levels of organization of physical processes inside neurons. At the microscale, the morphology of the rendered CA1 pyramidal neuron (NMO_00223) from rat hippocampus (http://neuromorpho.org) (accessed on 1 December 2023) reflects the functional specialization of dendrites and axon for the input and output of electric signals, respectively. At the nanoscale, the electric activity of neurons is generated by voltage-gated ion channels, which are inserted in the neuronal plasma membrane. The general structure of ion channels is illustrated with a single voltage-gated K^+^ channel composed of four protein α-subunits, each of which has six α-helices traversing the plasma membrane. The fourth α-helix is positively charged and acts as voltage sensor. At the picoscale, elementary electric charges within the voltage sensor could be modeled as qubits represented by Bloch spheres. For the diameter of each qubit is used the Compton wavelength of electron. Consecutive magnifications from micrometer (μm) to picometer (pm) scale are indicated by × symbol. Modified from Ref. [149].

In the quantum physical world, the sentience and free will possessed by the brain cortex are no longer a mystery as these physical properties are already inherent in all elementary physical particles (Figure 5). The quantum physicist Freeman Dyson expresses the latter fact candidly:

“Our consciousness is not just a passive epiphenomenon carried along by the chemical events in our brains, but is an active agent forcing the molecular complexes to make choices between one quantum state and another. In other words, mind is already inherent in every electron, and the processes of human consciousness differ only in degree but not in kind from the processes of choice between quantum states which we call “chance” when they are made by electrons” [148].

All biomolecular assemblies involve quantum interactions through strong covalent or weak hydrogen bonding at the nanoscale [150,151]. The quantum substrates present inside neurons support *thinking* as a quantum phenomenon [152,153,154]. Yet, one may wonder what the difference could be, e.g., between the electrons inside voltage-gated ion channels of electrically active neurons in living neural tissue (Figure 5) and the electrons inside inanimate matter, provided that the electrons are sentient by their quantum nature. The answer lies in the availability of *free energy*, which is continuously consumed by living organisms [155,156,157]. In particular, the continuous flow of metabolic energy allows for the creation of biologically ordered microenvironments [158] in which the dynamic evolution of quantum probability amplitudes of individual excitations remains localized in space [159,160,161,162]. This localization of excitations comprises a physical mechanism for biological storage and recollection of *memories* [1]. In other words, inanimate physical objects at thermal equilibrium may possess stochastic sentience, but everything that is experienced or felt would be *memoryless*. Presence of memories allow us to communicate with living brains, but not with rocks [3].

**Figure 6:**
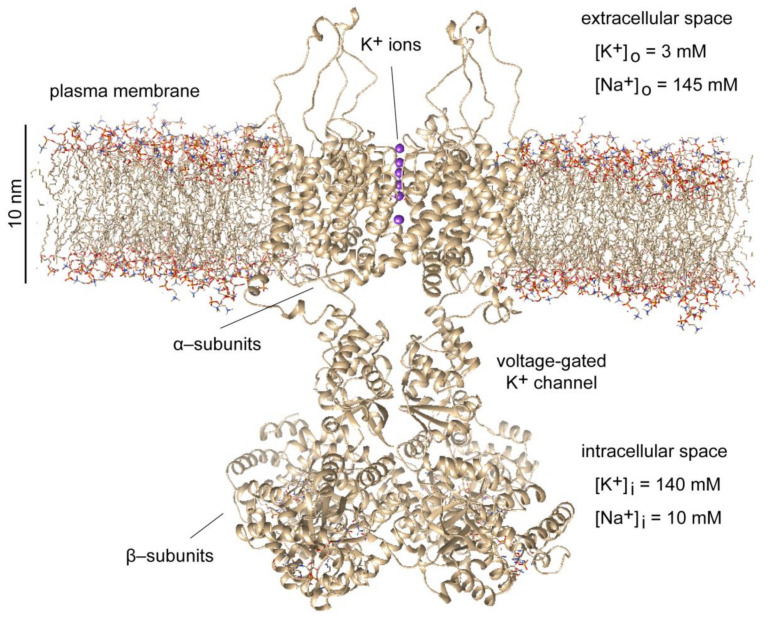
Molecular structure of full-length shaker Kv1.2 voltage-gated K^+^ channel from rat (*Rattus norvegicus*) composed of four α-subunits and four β-subunits incorporated into dipalmitoylphosphatidylcholine (DPPC) phospholipid bilayer. Free energy released by adenosine triphosphate (ATP) is used by Na^+^/K^+^-ATPase pump to establish different resting millimolar (mM) concentrations of K^+^ and Na^+^ ions on the inner side or on the outer side of neuronal plasma membrane. The phospholipid bilayer is hydrophobic and thus impermeable to ions, which are hydrophilic. The concentration gradient of K^+^ ions across the phospholipid bilayer acts as an electric battery that provides the energy for the generation of hyperpolarizing K^+^ electric current through the open voltage-gated K^+^ channels. The selectivity filter of the voltage-gated K^+^ channel contains a string of trapped K^+^ ions, which are allowed to move through the open channel pore only when the transmembrane voltage is depolarized toward more positive values compared to the physiological resting membrane potential of −70 mV. The 3LUT model from the Protein Data Bank (https://www.rcsb.org/structure/3LUT) (accessed on 1 December 2023) was visualized using the software UCSF Chimera ver. 1.11.2 (https://www.cgl.ucsf.edu/chimera/) (accessed on 1 December 2023).

A concrete molecular example of how neurons utilize Gibbs free energy to generate electric currents that serve as a form of *short-term memory* is provided in Figure 6. The free energy released through hydrolysis of adenosine triphosphate (ATP) molecules is used by neuronal Na^+^/K^+^-ATPase pump [163] to establish different concentration gradients of K^+^ and Na^+^ ions across the neuronal plasma membrane [93]. Inside the neuronal cytosol, the intracellular resting ion concentrations are [K^+^]_i_ = 140 mM and [Na^+^]_i_ = 10 mM, whereas in the extracellular space outside the neuron the resting ion concentrations are [K^+^]_o_ = 3 mM and [Na^+^]_o_ = 145 mM [164,165,166,167]. These transmembrane K^+^ and Na^+^ concentration gradients act as an electric battery that provides the energy needed for the generation of hyperpolarizing K^+^ electric current (i.e., K^+^ ions exit the neuron) through voltage-gated K^+^ channels or depolarizing Na^+^ electric current (i.e., Na^+^ ions enter the neuron) through voltage-gated Na^+^ channels. The selectivity of ion transmission, such that a particular type of voltage-gated ion channel conducts only a particular type of ions, is ensured by genetically encoded amino acid composition of the protein α-helices that line the narrowest part of the channel’s open pore in the so-called *selectivity filter*. In the case of Kv1.2 voltage-gated K^+^ channel, the selectivity filter contains a string of trapped K^+^ ions (Figure 6), which are reminiscent of the operation of experimental quantum computers based on trapped ions [168,169,170], even though the biologically trapped K^+^ ions are eventually allowed to move through the open channel pore and the neurons operate at physiological temperature of ≈300 K [171].

The quantum nature of voltage-gated ion channel gating is manifested in single-channel patch clamp recordings as stochastic transitions of the channel between open and closed states [172], which are characterized with certain expected steady-state conductivity of the channel, typically represented by a sigmoid curve at different values of the transmembrane voltage of the neuron. This expected steady-state conductivity of the voltage-gated ion channel is reached dynamically with a characteristic time constant [173,174,175]. For certain voltage-gated ion channels that can undergo both activation and inactivation, there are multiple gating variables, each of which could have its own time constant [176,177,178]. Neuronal electric activity could also trigger cascades of biochemical reactions, such as phosphorylation or dephosphorylation of ion channels, which could modify the ion channel time constants and thereby prolong the time period during which the short-term memory is kept active.

Recent quantum simulations of voltage-gated K^+^ channels, using density functional theory (DFT) with hybrid B3LYP functional on a supercomputer, have confirmed that quantum dynamics, including quantum tunneling through classically forbidden potential energy barriers, is indeed indispensable for the proper understanding of ion channel gating and selective conductivity [179,180,181,182]. For example, it was demonstrated that in the closed state of the voltage-gated K^+^ channel, a transient ice nanocrystal formed of four water molecules is able to occlude the channel pore [182]. This kind of water freezing at the nanoscale poses a challenge to the naïve classical view of the cellular cytosol as a warm, chaotic “liquid soup” of chemicals.

The *free energy principle* is a physical foundation to the evolution of morphogenetic structures from individual biomolecules to cells, tissues, organs, and organisms [158]. Motivated by how an entirety of simple organisms, extending all the way up to complex animals, possess multiple cognitive faculties for observing and acting upon their environments and do so in a context-sensitive way [183], the aspect of free energy was established in an information flow, scale-free architecture for generic quantum systems in terms of *quantum reference frames* following the formalism of hierarchical Bayesian inference [154]. When applied to neuronal systems, the quantum reference frame approach describes how neurons exchange information with their environments via physical interactions in terms of measurement and manipulative action [184,185].

The organization of pluripotent sentient cells into fully differentiated neural networks capable of supporting animal consciousness, has been informed by recent discoveries in embryology [186,187,188], histology [189], and anatomy [190,191,192]. Growing of brain organoids in vitro could also help the development of novel treatments for neurological disease [193,194,195] and pave the way toward creation of artificial consciousness [196,197,198]. All this cutting-edge research on consciousness would benefit from further theoretical and computational studies of quantum activity in functional biomolecules, due to their manifestation of sentience and free will.

## 6. Conclusions

Conscious experiences are our only means of accessing and comprehending the surrounding physical world. Because the conscious experiences are not directly observable in others [199,200,201], psychologists have often relied on elicited behavioral responses in order to decide whether a physical agent is conscious or not [202,203,204]. This behaviorist approach, however, runs into insurmountable problems in the framework of classical physics because the mathematical properties of ordinary differential equations do not allow for causal potency of emergent conscious experiences, whereas the deterministic dynamics of classical physical quantities, such as mass, charge, length and time, provides no easy reductive identification of consciousness with a physical entity that is capable of making choices from a set of available future courses of action [29]. Consequently, the evolution of human consciousness is utterly inexplicable from the principles of classical physics and some philosophers have prematurely declared that consciousness is nonphysical.

Fortunately, classical physics was already experimentally discredited at the end of 19th century due to its inability to explain different physical phenomena, including the blackbody radiation [75,76,77,205,206,207,208,209,210,211], photoelectric effect [78,79], stability of atoms [212,213,214], and hydrogen spectrum [215]. The newly discovered quantum principles were revolutionary because they not only predicted correctly experimental observations, but also endowed the physical reality with capacity to choose among different quantum physical potentialities with actualization of some of them thereby irreversibly changing the future history of the universe. This incorporation of sentience and free will in the quantum fabric of physical reality removes all traces of mystery about the evolution of consciousness in animals and reassures us that we live inside a hospitable universe where our conscious choices do make a difference through causal action upon the physical world [148].

Darwinian theory of biological evolution by natural selection [63,64] explains the great diversity of living organisms on Earth and relates them to a common ancestor that appeared 3.8 billion years ago. Through genetic methods we are capable of reconstructing the metabolism of the last universal common ancestor and may even describe the habitat in which it resided as a geochemically active environment rich in H_2_, CO_2_ and iron [216]. The evolutionary changes of living organisms, however, do not violate physical laws, which means that sentience and free will cannot be miraculously irrupted into an insentient physical world. Giving up the false and harmful idea that there was a “spark of consciousness” that separates us humans from other beasts [37], the evolutionary theory could be divorced from the discredited classical physics and put on a stable foundation comprised of quantum physical laws. In quantum physics, consciousness is causally effective and capable of making genuine choices for control of observed behavior [29]. The transition from classical to quantum thinking in biological sciences could be enabled by appreciating the quantum nature of physical systems as a useful physical resource that allows them to achieve tasks that are impossible for classical systems [217,218,219]. The rapid progress achieved by quantum information science and technology in recent decades is accompanied by a significant increase in the available introductory literature on the subject [144,220,221,222], which could help more biological researchers join the exploration of the fascinating interdisciplinary field of quantum biology [171].

## 7. Glossary

***Awareness*** is the cognitive state of knowing and understanding that something is happening or exists. Self-awareness is the act of comprehending our own existence.

***Consciousness*** is the single, seamlessly unified, subjective, phenomenological, first-person point of view of our mental states, experiences or feelings.

***Darwinian evolution*** is a natural process of descent with modification of living organisms through which biological species change over time, give rise to new species, and share a common ancestor.

***Epiphenomenon*** is a phenomenon or an entity which does not have any causal powers in the physical world.

***Free will*** is the inherent capacity of physical agents to perform genuine choices among at least two available future physical outcomes.

***Physical*** is anything that exists either as an entity or as a property of existing things inside the universe. Nonphysical is anything that does not exist in the universe. 

***Self-recognition*** is the ability to recognizing oneself as separate from others. Animal self-recognition is usually confirmed by the use of a mirror to touch and/or investigate normally unseen parts of one’s own body.

***Sentience*** is the capacity to experience or to feel. Elementary sentient units could be part of a single conscious mind. However, any collection of multiple individual conscious minds, such as the population of a city, is not sentient because such collection as a whole does not have its own single conscious mind.

***Universe*** is the collection of all existing things.

## Data Availability

Reconstructions of hippocampal pyramidal CA1 neurons are freely available from http://neuromorpho.org.
